# A Strictly Unsupervised Deep Learning Method for HEp-2 Cell Image Classification

**DOI:** 10.3390/s20092717

**Published:** 2020-05-09

**Authors:** Caleb Vununu, Suk-Hwan Lee, Ki-Ryong Kwon

**Affiliations:** 1 Department of IT Convergence and Application Engineering, Pukyong National University, Busan 48513, Korea; exen.xmen@gmail.com; 2 Department of Computer Engineering, Dong-A University, Busan 49315, Korea; skylee@dau.ac.kr

**Keywords:** HEp-2 cell images classification, computer-aided diagnosis, pattern recognition, deep learning, convolutional autoencoders, cell images clustering

## Abstract

Classifying the images that portray the Human Epithelial cells of type 2 (HEp-2) represents one of the most important steps in the diagnosis procedure of autoimmune diseases. Performing this classification manually represents an extremely complicated task due to the heterogeneity of these cellular images. Hence, an automated classification scheme appears to be necessary. However, the majority of the available methods prefer to utilize the supervised learning approach for this problem. The need for thousands of images labelled manually can represent a difficulty with this approach. The first contribution of this work is to demonstrate that classifying HEp-2 cell images can also be done using the unsupervised learning paradigm. Unlike the majority of the existing methods, we propose here a deep learning scheme that performs both the feature extraction and the cells’ discrimination through an end-to-end unsupervised paradigm. We propose the use of a deep convolutional autoencoder (DCAE) that performs feature extraction via an encoding–decoding scheme. At the same time, we embed in the network a clustering layer whose purpose is to automatically discriminate, during the feature learning process, the latent representations produced by the DCAE. Furthermore, we investigate how the quality of the network’s reconstruction can affect the quality of the produced representations. We have investigated the effectiveness of our method on some benchmark datasets and we demonstrate here that the unsupervised learning, when done properly, performs at the same level as the actual supervised learning-based state-of-the-art methods in terms of accuracy.

## 1. Introduction

Computer-aided diagnosis (CAD) refers to all systems that aim to cement the efficiency and automation of disease diagnostic procedures with the help of methods such as machine learning techniques. As far as autoimmune diseases are concerned, the analysis of the Human Epithelial of type 2 (HEp-2) cell patterns is one of the most important steps of the diagnostic procedure [1]. This analysis includes the classification of the different types of HEp-2 cells. Performing this analysis manually represents a relatively complicated work due to the complexity exhibited by these cellular patterns. Hence, developing CAD systems capable of helping pathologists to fully perform this analysis is necessary. 

In case of the diagnosis of autoimmune diseases, one of the most important steps in CAD systems is the automatic classification of the images representing the different HEp-2 cell types [2]. During the past decade, researchers have proposed numerous different methods for the automatic classification of HEp-2 cellular images. As a normal pattern recognition problem [3], HEp-2 cell image classification methods comprise mainly two distinctive steps: feature extraction and discrimination (referred also as classification). Moreover, two different approaches can be separated in the literature: conventional machine learning methods based on handcrafted features and the deep learning methods based on the automatic feature learning approach. 

Methods based on the conventional machine learning scheme typically present a certain feature extraction approach followed by a certain classification model. Some of the first works to have utilized the conventional handcrafted features include the method proposed by Cataldo et al. [4], who adopted the use of the gray level co-occurrence matrix and the discrete cosine transform (DCT) as the features for classifying HEp-2 cell images. Wiliem et al. [5] used the codebooks that were generated from the DCT features and from the scale-invariant feature transform (SIFT) descriptors. Both works utilized the support vector machine (SVM) as the classifier. Nosaka et al. [6] adopted the texture-based features known as the local binary patterns (LBP) and also used an SVM in order to perform the classification process. Huang et al. [7] proposed using two kinds of features at the same time: the textural and the statistical features. They constructed a vector that mixes the two sets of features and used that hybrid vector as the input of a self-organizing map (SOM) for the final discrimination step.

Thibault et al. [8] proposed using another kind of statistical feature called the gray-level size zone matrix. In their work, the nearest-neighbor classifier was employed in order to perform the discrimination of the extracted statistical features. Another work by Wiliem et al. [9] utilized the same statistical feature proposed in [8]. Xu et al. [10] proposed using the linear local distance coding technique for building the feature vectors. These features were then utilized as the inputs for training an SVM.

As we remarked in [7], the construction of hybrid feature vectors, which consists of mixing different kinds of features in the same vector in order to capture the different information of the cells at the same time, is widely used in this field. In fact, the inhomogeneity exhibited by the HEp-2 cell image datasets encourages the mix (hybridization) of different kinds of features in order to seize the different meaningful characteristics of the cells. We can see such hybridization in the method proposed by Cataldo et al. [11]. Here, the authors built a complex hybrid feature vector by combining the morphological features and the rotation-invariant Gabor features [12]. They went further in the hybridization by using, in addition to the features previously cited, different kinds of LBP descriptors, such as rotation-invariant uniform LBPs [13], co-occurrence adjacent LBPs [14], completed LBPs [15] and, finally, rotation-invariant co-occurrence of adjacent LBPs, which were also used as the principal features in [6].

Another kind of hybridization was adopted in the method proposed by Theodorakopoulos et al. [16]. In this hybrid vector construction, the authors combined the already mentioned LBP and SIFT descriptors and utilized the obtained hybrid feature vector for also training an SVM. Many different handcrafted feature-based methods can be found in [17,18]. Foggia et al. [3] undertook an interesting review of many other handcrafted features proposed for this task.

As can be seen thus far, the performance of these conventional machine learning methods exclusively depends on the efficiency of the chosen features. With these methods, the final precision of the CAD systems is typically based on the subjective choice of the features. It is necessary to note that even though the accuracy of the methods based on the conventional handcrafted features has been, and can still be, improved, these methods have been abandoned in favor of the deep learning-based methods, which, in fact, permit feature learning in an automatic fashion.

Methods using deep learning [19] have been widely utilized by researchers for different topics. They have demonstrated their effectiveness by considerably improving the results obtained in object recognition tasks [20,21]. They have also been utilized in the HEp-2 cell image classification topic. Convolutional neural networks (CNNs) have the advantage of affording a feature learning process that can be undertaken in an automatic way. More importantly, they provide far better results when compared to the handcrafted features. Foggia et al. [2] were among the pioneers to utilize CNN in order to classify the different types of HEp-2 cells. This work was presented during the 2012 edition of the international conference on pattern recognition (ICPR) HEp-2 cell classification contest. Even though the results obtained in [2] were remarkable, the datasets that were available at that time were not diversified enough. In fact, one characteristic of deep learning is the need of having such a large amount of training examples in order to allow the network to be efficient. 

A large number of improvements have since been made to the datasets. Gao et al. [22] proposed a simple CNN model for classifying different HEp-2 cell images. In this work, the authors were among the first to use many different data augmentation techniques for this topic. Li et al. [23] proposed the combination of the ideas used in two of the most popular CNN models: the residual short-cut technique utilized by the ResNet [24] and the network-in-network technique, also called “Inception”, utilized by the GoogleNet [25]. They labeled their obtained module as deep residual inception (DRI). Phan et al. [26] performed simple transfer learning using a CNN model that was pre-trained on the ImageNet dataset.

While the DRI module-based network in [23] represents one of the actual state-of-the-art methods for the classification of HEp-2 cell images, another can be found in the transfer learning method proposed by Lei et al. [27]. In this work [27], the authors proposed adopting a modified version of the ResNet [24] and used it in two levels: in the first step, the network is pre-trained with a small dataset of the HEp-2 images and the obtained trained network is fine-tuned with another big dataset. Their approach was named cross-modal transfer learning. Another state-of-the-art method can be found in the work by Shen et al. [28] where the authors also utilized the ResNet by modifying the residual connections between the layers, with the so-called deep-cross residual (DCR) module. Note that they also mixed different data augmentation techniques. Two other state-of-the-art methods are outlined in [29,30].

The most important point to note about these deep learning-based methods is that they all utilize the supervised learning paradigm. The supervised learning approach has the exigency of having images labelled manually by experts in order to train the network. This can represent a drawback for these methods, knowing that deep learning structures necessitate thousands of images. Labelling this quantity of images manually can represent a challenging and burdensome task. On the other hand, the unsupervised learning paradigm presents the advantage of performing the feature extraction without the need of any labelled data during training.

In our previous work [31], we investigated an unsupervised learning scheme for HEp-2 cell classification. However, only the feature extraction part was completely undertaken in an unsupervised learning way. The created features were given to a nonlinear classifier that learned with the use of the manually labeled data. Thus, while the feature extraction was undertaken in an unsupervised way, discrimination was still required to be supervised. The first contribution of the present work is to propose a scheme where the feature extraction and the discrimination parts are both performed in a strictly unsupervised paradigm.

We adopt the deep convolutional autoencoder (DCAE) as the principal feature extractor. The DCAE takes the original images as inputs and learns how to reproduce them via an encoding–decoding structure. Unlike in our previous work, where the features learned by the DCAE were extracted and then utilized as the inputs of a nonlinear classifier, we propose to embed a clustering layer in the DCAE. The clustering layer will learn, in every single step of the training process, how to automatically discriminate the latent representations produced by the DCAE. 

The idea of embedding a clustering process inside a deep neural network (DNN)-based structure was used, in different manners, by Yang et al. [32] and Guo et al. [33], the first for the clustering of randomly generated data and the latter for the discrimination of images representing handwritten digits. The DNN used in [32] was the stacked autoencoders (SAEs), while the authors in [33] also utilized the DCAE. 

Another approach for performing clustering in DNN was proposed by Caron et al. [34], where the authors connected a clustering module to the output layer of a CNN. In their work, the CNN’s output layer was utilized in a different manner. Instead of computing the difference between the CNN’s output and the input’s label as is usually done in supervised learning, the authors proposed computing the difference between the CNN’s output and what they have called the pseudo-labels. These pseudo-labels are just the input’s cluster assignments computed using the k-means [35] cost function. Their method alternately computes the cluster assignments with the k-means using the output values of the CNN and subsequently updates the CNN’s parameters by utilizing these cluster assignments as the labels. They adopted the AlexNet [21] and the VGG-16 [36] as their networks. In this work, we have adopted the learning function utilized by Yang et al. [32], which also incorporates the k-means clustering idea in the learning process of the DNN. However, instead of using the SAEs, as is done in [32], or the CNN, as proposed in [34], we adopt the use of the DCAE, as proposed in [33]. While the DCAE will perform the feature extraction, the clustering layer will learn dynamically how to discriminate these features, which avoids the need of having any labelled images during the training.

Unlike in [33], where the efficiency of the reconstruction process performed in the DCAE’s decoder is assured only by the model’s loss function, the second contribution of the present work is to utilize some techniques in order to minimize the loss of the spatial information of the original input images and, thus, to ensure a certain preservation of the local structure of the original pixels, which will probably ensure a better reconstruction. In fact, the down-sampling process incorporated in the DCAE and performed by its encoder causes the loss of the spatial details inside the network. We investigate in this work how the quality of the reconstructed images can affect the quality and, thus, the discrimination potentiality, of the latent representations learned by the DCAE.

**Figure 1:**
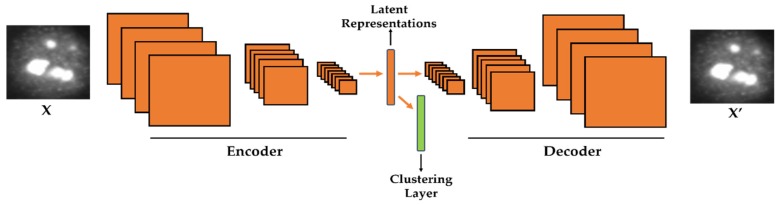
Illustration of the proposed method. A clustering layer is embedded in the convolutional autoencoder in order to learn how to cluster the latent representations. X is the original cellular image and X′ is the reconstruction.

We investigated the effectiveness of the proposed deep clustering method on two benchmark datasets, the ICPR 2016 dataset [37] and the SNPHEp-2 Cell dataset [5]. The obtained results demonstrate that the proposed strictly unsupervised learning method far outperforms the handcrafted features and performs at least at the same level as the state-of-the-art supervised deep learning methods. The schematic illustration of the proposed method is shown in Figure 1. The remaining content of the paper is organized as follows. The next section presents in detail each step of the proposed scheme. Section 3 discusses the experimental setup and details, presents the obtained results, and also addresses a detailed comparative study with the actual state-of-the-art deep learning-based supervised learning methods.

## 2. Proposed Clustering Method

### 2.1. Feature Learning with the Convolutional Autoencoder

Autoencoders [38,39] refer to unsupervised learning-based structures that are mainly used for the purpose of dimensionality reduction. Dimensionality reduction, which can also be utilized as a feature extraction, consists of finding a better representation of the data in lower dimensions. DNN-based autoencoders consist of two principal structures: the encoder and the decoder. Given an input signal **x**, with $x\in R^{D}$, the encoder takes it and transforms it into a contracted representation **y**, with $y\in R^{d}$, with d<D, by utilizing the transformation function g in such a way that

(1) y=g(θx),

where θ englobes all the different parameters of the encoder, which can be a set of weights and biases. After the encoder transforms the input signal **x** into **y** using Equation (1), the decoder takes the contracted representation **y** as input and uses the same transformation function g but, in this time, for the purpose of reconstructing the original signal **x**. Here, let **z** be the output of the decoder. Then, we have

(2) $$z=g\left(\theta^{\prime }y\right),$$

where θ**′** englobes all the different parameters of the decoder, which can also be a set of weights and biases. Finally, the network, composed of both the encoder and the decoder, should learn the parameters θ (encoder) and θ**′** (decoder) in such a way that the reconstructed signal **z** equals the input vector signal **x**. This means that the network should learn the parameters that minimize as much as possible the existing differences between the input **x** and the final network’s output **z**.

In case of images or signals that are represented in a two-dimensional (2-D) fashion, this described encoding–decoding process can be realized with the use of another DNN-based structure, the so-called deep convolutional autoencoder (DCAE). Because it takes 2-D signals, the DCAE is likely to be more efficient than the SAEs as far as images are concerned. In the DCAE, the encoder performs the down-sampling process, while the decoder will perform the opposite operation, the up-sampling (see Figure 1 for the illustration of the encoding–decoding process). The down-sampling can be realized with the use of the convolutional and/or pooling layers. On the other hand, the up-sampling is done by the backwards convolution (transposed convolution), often called “deconvolution”, and/or by the backwards pooling, often denoted as “unpooling” operations. The final solution of the DCAE is given by

(3) $$\left(\theta ,\theta^{\prime }\right)=\underset{\theta ,\theta^{\prime }}{\mathrm{argmin}}L\left(xz\right),$$

where **z** denotes the reconstruction (decoder’s output), **x** represents the original image (encoder’s input), and the function L(·) represents the cost function that measures the differences between **x** and **z**. In this work, the adopted cost function is the squared Euclidean distance described as

(4) $$L\left(xz\right)=\overset{N}{\underset{i=1}{\sum }}{\left||x_{i}-z_{i}|\right|}_{2}^{2}$$

where the value *N* represents the total number of images. The network will learn the parameters θ and θ**′** by minimizing the cost function represented in Equation (4).

### 2.2. Embedded Clustering Layer for the Convolutional Autoencoder 

Some of the early works that aimed to incorporate the clustering process in the DNN can be found in the methods proposed by Xie et al. [40] and Yang et al. [41]. Their idea was to connect a clustering module to the output layer of a DNN and to learn the DNN’s parameters and the clusters’ centers at the same time. In their works, only the clustering loss is used during the parameters’ updating process, as the reconstruction is not a concern. In addition, the fact that they have adopted the plain network, the SAEs, which takes one-dimensional data as inputs, poses the problem of the preservation of the local structure of the pixels of the original images. Guo et al. [33] proposed to use the DCAE, instead of the SAEs, with the clustering layer incorporated in the middle of the network, instead of connecting it to the output layer, as done in [40,41]. However, in their work, the reconstruction problem is solved only by adding a reconstruction loss to the clustering loss. In our work, as we will explain in the next section, in addition to using a reconstruction loss, we also apply some techniques that can assure a better reconstruction and investigate how it can affect the quality of the latent representations.

Yang et al. [32] utilized the SAEs and embedded a clustering module (or layer) in the middle. They used a global loss function that incorporates the reconstruction and the clustering losses. In their work, the k-means clustering cost is used as the clustering loss. In general, the global loss function *L* for the DCAE can be expressed as

(5) $$L=L_{r}+\gamma L_{c},$$

where $L_{r}$ is the reconstruction loss and $L_{c}$ denotes the clustering loss. The parameter γ is used to balance the importance of the clustering loss in the global loss. If γ is greater than one, the clustering loss will have a greater effect on the global loss, otherwise, the reconstruction loss will have more incidence during training. The reconstruction loss $L_{r}$ is defined to minimize the differences between the output of the decoder, which is the final output of the network, and the original input image. This function is defined, in our case, in Equation (4). In addition, using the k-means approach, the clustering loss can be defined as

(6) $$L_{c}=\overset{N}{\underset{i=1}{\sum }}{\left||x_{i}-Cy_{i}|\right|}_{2}^{2},$$

where the constant *N* denotes the number of data points. $y_{i}$ is a *k*-dimensional vector representing the cluster assignment vector of the input $x_{i}$. Note that all the elements of $y_{i}$ are zero except the element corresponding to the cluster index of the input, whose value is 1. **C** is a *M*×*k* matrix, with *M* being the dimension of the input $x_{i}$ and *k* representing the number of the clusters. The matrix **C** contains the clusters’ centroids that must be learned. Note that the centroids have the same dimension as the input data. Minimizing Equation (6) can be thought of as solving the following problem:

(7) $$\underset{\left(C\;\in \;R^{M\times k}\right),\;\left(y_{i}\;\in \;R^{k}\right)}{\min}\overset{N}{\underset{i=1}{\sum }}{\left||x_{i}-Cy_{i}|\right|}_{2}^{2}\;\;\mathrm{such}\;\mathrm{that}\;y_{j,\;i}\in \left\{0,1\right\},\;y_{i}{}^{T}1_{k}=1,$$

where $y_{j,\;i}$ represents the elements of the assignment vector $y_{i}$, with *j* varying from 1 to *k.* $1_{k}$ represents a *k*-dimensional vector containing only the values 1. Note that with $y_{i}{}^{T}1_{k}=1$, the condition that every element of $y_{i}$ should be zero except one element, whose value should be 1, is satisfied. 

In our situation, the input **x** in Equations (6) and (7) is precisely the latent representations (features) learned by the DCAE, as opposed in [34], where the input **x** represents the output of the CNN. This means that, at iteration *t* of the training process, the clusters’ centroids contained in the matrix **C** and the clusters’ assignments are updated according to the latent representations **x** produced by the DCAE at the same iteration.

By simultaneously minimizing the reconstruction and the clustering losses, we can expect two important effects for our approach. Firstly, we expect that the DCAE will learn to produce features that are k-means-friendly, which means that the feature space produced by our network is expected to have the property of being easily separated by distinctive clusters. Secondly, the computed centroids at each iteration will be forced to follow the distribution of the features, which reinforces the probability of producing distinctive clusters.

### 2.3. Reconstruction Consistency 

The architecture of the proposed DCAE is shown in Table 1. We can distinguishably notice the two parts of the network: the encoder, which reduces gradually the spatial size of the input (see the 6th column) while increasing the volume’s depth (see the 3rd column, the number of feature maps); and the decoder, which gradually increases the spatial size of the input while decreasing the depth. Note that the size of the input image is 112 × 112.

One important drawback of the DCAE structure is the down-sampling process performed by the encoder. The input image is systematically down-sampled while we progress inside the network until we arrive at the latent representations’ space, after which the decoding, which means the up-sampling, begins. This has as the consequence that the network loses the spatial information of the image layer after layer. By decreasing the size of the image, the local structure of the pixels is also distorted. This distortion complicates the reconstruction process. That is the reason why the preservation of the local structure of the original pixels can be essential. Using the reconstruction loss, as opposed to the fact of using only the clustering loss [40,41], can be the solution to this problem, as discussed by Guo et al. [33]. Instead, we consider that, if incorporating the reconstruction loss in the global loss of the network, as defined in Equation (5), can help in that direction, it is not the only way of assuring a better preservation of the spatial structure of the original pixels.

One of the techniques that can also help to minimize the loss of the cues related to the spatial structure of the data can be the fact of avoiding the use of big filters. As we can see in Table 1, all the convolution operations utilize a 3 × 3 filter size. Only the last convolutional layer from the encoder (Conv 5) utilizes a different filter size (7 × 7). Note that the 7 × 7 filter is used in order to produce the one-dimensional features’ layer that follows. Note also that the stride and padding, except for Conv 5, are used in such a way that the output of every convolutional layer has the same spatial extent with its input. That is, the convolution operations do not alter or distort the spatiality of the input. This property also attenuates to a certain level the loss of the information related to the spatiality in the encoder. The down-sampling operation is realized exclusively by the pooling layers. We can see, in Table 1, how every output volume of the pooling layer is spatially down-sampled by half. The layer in the middle of network can be thought of as a one-dimensional vector containing 512 elements. This layer, as we can see, contains the features of the DCAE that will be passed to the clustering layer in order to compute the clusters’ centroids.

Directly after the 1 × 1 × 512 feature vector, the up-sampling mechanism in the decoder starts with the stacking of many convolutional and unpooling layers until we reach the original size. Note that we did not use the transposed convolution operations, except for the “Conv 6”, which is the only transposed convolutional layer of the network precisely because it must increase the size of the 1 × 1 × 512 feature vector. All the remaining layers, from “Conv 7” to “Conv 10”, apply normal convolution operations. Therefore, every remaining up-sampling process is performed only by the unpooling layers, which are the opposite correspondents of the pooling layers. In Table 1, the unpooling layers are depicted as “Unpool n”. Here also in the decoder, the convolution operations do not alter the spatial dimensions of the inputs. 

**Figure 2:**
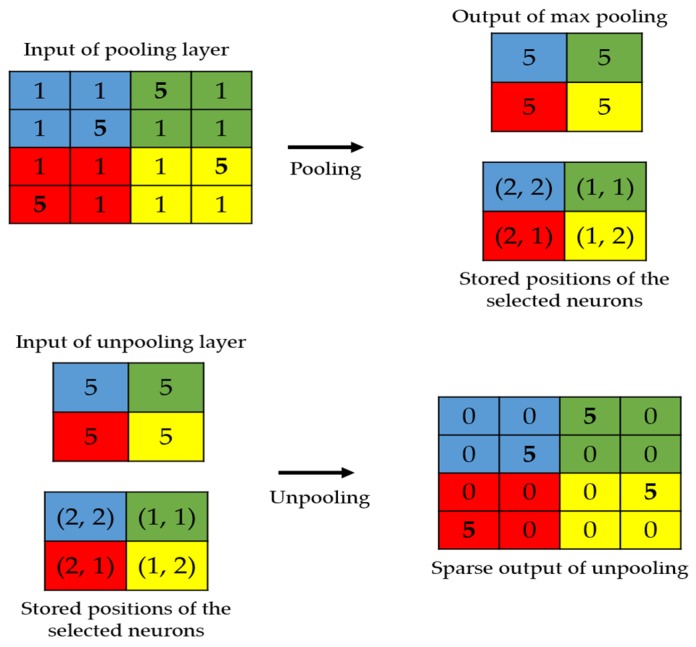
Illustration of the storage technique. The positions of the strongest activations are utilized in order to produce the sparse output.

The local structure preservation problem has also been faced in the segmentation topics, where the original image’s spatial information is critical [42,43]. We propose here to apply two techniques used in the segmentation’s problems. The first is to use the position storage technique, as proposed by Badrinarayana et al. [42]. This technique, as illustrated in Figure 2, consists of storing the positions of the selected activations during the maximum pooling process performed in the encoder. In the decoder, the unpooling process will exclusively consist of placing the activations at the stored positions and setting all the remaining values to zero.

**Figure 3:**
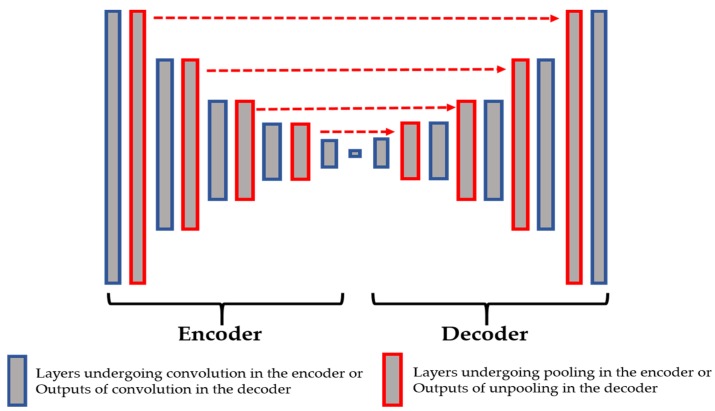
The connections between the pooling layers from the encoder and the unpooling layers from the decoder.

In Figure 3, we show the connections between the layers from the encoder and the decoder. The layers shown in red in the encoder are those that undergo the maximum pooling operation. While applying the maximum pooling, we store the positions of the strongest activations in the feature map. Note that the strongest activation here refers to the biggest value inside the 2 × 2 filter of the max pooling, as clearly depicted in Figure 2. On the other hand, the layers shown in red in the decoder are the outputs of the unpooling operations and they are made by using the stored positions from their corresponding layer in the encoder. All of these layers are filled with the sparse-kind outputs depicted in Figure 2. As we can see in Figure 3, every output of the unpooling layer goes through a convolution layer whose purpose will be, indeed, to densify the sparse representations. By using this storage technique, the network does not change and the architecture remains similar to the one depicted in Table 1.

**Figure 4:**
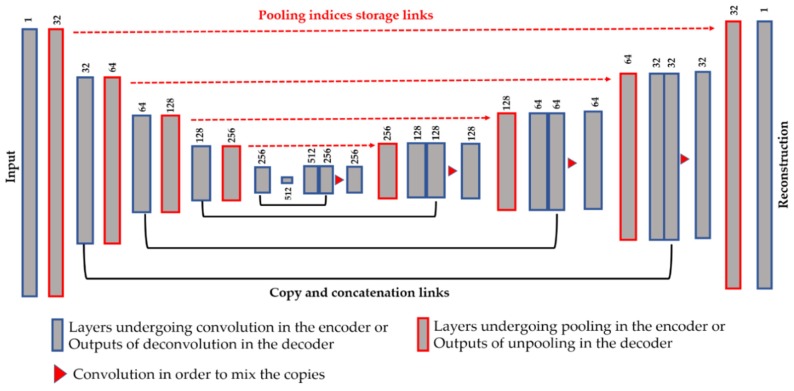
Illustration of the proposed network using the storage and copy/concatenate techniques at the same time. The dimensions shown above each layer refers to the number of features maps (the depth of the volume). See Table 1 for the spatial dimensions of each volume.

Another technique in order to assure a better preservation of the local structure is the use of the copy and concatenation operations, as proposed by Ronneberger et al. [43] with the so-called Unet, for the segmentation of the cellular images. This idea consists of mixing, by concatenation operations, the features from the encoder and their corresponding features in the decoder. All of the connections are made before the down-sampling process. Figure 4 illustrates the copy and concatenate process and provides the details of the finally proposed network. We propose using the pooling indices storage and the copy-and-concatenation techniques at the same time (see Figure 4 for the final structure). The unpooling is done in the same way as described above.

From Figure 4, we can remark on the three main differences with the Unet. First, we did not use the transposed convolution, except for “Conv 6” layer. All of the feature extraction process is performed by the convolutional layers and the nonlinearities from the ReLu [38]. Second, the up-sampling operation is exclusively performed by the unpooling layers using the pooling indices storage technique. Third, as expected, the final layer of our network comprises one single channel and represents the reconstruction of the original images, and not the segmentation mask. As we can see in Figure 4, after every copy-and-concatenation process, we apply a convolution operation, represented by the red triangle in Figure 4, whose purpose is precisely to mix up the information from the encoder and the decoder. When the copy-and-concatenation process increases the depth of a volume, the following convolution will combine the information and permit returning to the original volume’s depth. The network shown in Figure 4 has a similar structure to that in Table 1, the only difference being the copy-and-concatenation layers. Next, we will discuss how these techniques can affect the quality of the features and, thus, the quality of the learned clusters.

## 3. Results

### 3.1. Experimental Setup and Dataset

**Figure 5:**
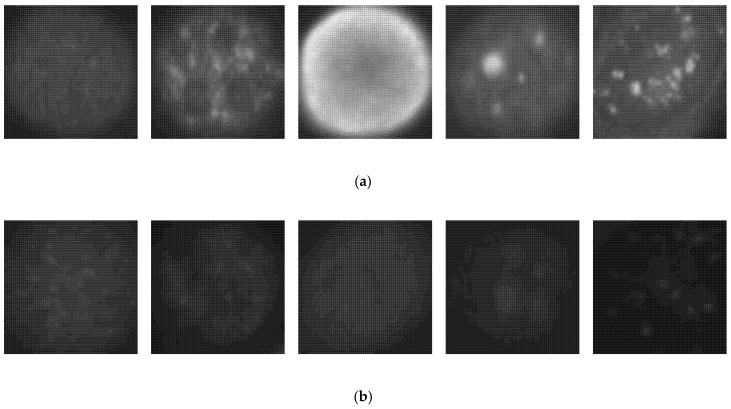
Example images from the SNPHEp-2 dataset. (**a**) The positive fluorescence illumination images. (**b**) The negative fluorescence illumination images. In (**a**) and (**b**), from the left to the right: homogeneous, coarse speckled, fine speckled, nucleolar and centromere cells.

We first show the results obtained using the SNPHEp-2 dataset. The description and the download link of this dataset can be found in [6]. The SNPHEp-2 dataset comprises five types of cells: homogenous cells, coarse speckled cells, fine speckled cells, nucleolar cells, and centromere cells. This dataset contains two levels of fluorescence intensity: positive and negative intensities. Some examples from the dataset are depicted in Figure 5 and we can observe that the different fluorescence intensities increase the intra-class variations of the dataset. In Figure 5a, we have the positive illumination images and Figure 5b shows the negative (or intermediate) illumination images. We can observe how the differences between the images belonging to the same cellular type but having different levels of fluorescence illumination are obvious, demonstrating the intra-class variations.

The dataset contains 1884 HEp-2 cell images. The images were all extracted from 40 different cell specimens. From the 40 specimens, 20 were used for the training sets and the remaining 20 were used for the testing sets. In total, there are 905 and 979 cell images for the training and testing sets, respectively. Each set (training and testing) contains five-fold validation splits of randomly selected images. In each set, the different splits are used for cross-validating the different models, each split containing 450 images approximatively. During the experiments, the images were upscaled to 112 × 112 in order to use them in our network. In order to improve the learning capability of the network, data augmentation was applied over the dataset. In every splitting set of the training data, the cells were rotated by 360° in steps of 18°, as done in [22,28]. Thus, the original training set was expanded by a factor of 20, a 360° quadrant containing 20 portions of 18°. We found that augmenting the training set greatly improves the accuracy over the testing set. All the results shown subsequently are those obtained after applying data augmentation in this form. 

As explained in the previous section, the global loss, defined in Equation (5), is minimized by updating the network’s parameters. In addition to the weights and biases usually associated with the DNN, we have the clusters’ centroids, which also need an update at every epoch during the training. As usually done with the k-means process, initial centroids are needed in order to launch the updating process. Instead of a random setting, we use a pre-training of the DCAE in order to generate the first clusters, as done in [32,33,40,41]. The idea is to firstly use the DCAE for generating a preliminary distribution of the data, and then, utilize the computed features for finding the initial centroids. This pre-training can be seen as setting γ to be zero in Equation (5), which means that the learning is done using only the reconstruction loss $L_{r}$. After generating the initial centroids, we perform the learning as proposed here using the global loss with γ>0. This second training process using the global loss is subsequently referred as the “global learning”. 

As explained in the previous section, the parameter γ is very important because it balances the importance of the clustering loss $L_{c}$ in the global loss. As also previously mentioned, the reconstruction loss itself can help to preserve the local structure of the data. Hence, as explained in [33], it is preferable that the coefficient γ is less than 1 in order to permit $L_{r}$ to have more importance than $L_{c}$ in the global loss. In our experiments, by using cross-validation, the best results were obtained with the value of γ being 0.1. Note that this value of γ provides also the best results in [33] when dealing with images. 

The parameters are updated by using back-propagation [44]. The gradients of the global loss can be derived as

(8) $$\frac{\partial L}{\partial \left(Z,\;c\right)}=\frac{\partial L_{r}}{\partial \left(Z,\;c\right)}+\gamma \frac{\partial L_{c}}{\partial \left(Z,\;c\right)},$$

where *Z* represents the set of weights and biases, and *c* represents the clusters’ centroids. Except for γ, for which different values (0 and 0.1) were used for the pre-training and the global learning, all the other hyperparameters were used similarly for the two steps. The learning rate was set to be 0.01 and the size of the mini-batch was 128. The momentum was set to be 0.9 and the weight initialization follows the process presented by He et al. [45]. The pre-training was done with 200 epochs, while the global learning was trained for 450 epochs. The experiments were done with the use of MATLAB R2019b and performed on a computer with a Core i7 3.40 GHz processor and 8 GB of RAM. A GPU implementation was used with a NVIDIA GeForce GTX 1080 Ti with 11,264 MB of memory.

Specific metrics are usually adopted for evaluating the clustering performance. These metrics include the normalized mutual information [46], the adjusted Rand index [47], and the clustering accuracy (ACC) [46]. Because we aim to compare our method with the state-of-the-art HEp-2 cell classification methods that all utilize supervised learning, we only use the ACC for showing the results, since it is equivalent to the classification accuracy measured in supervised learning. Since we have the actual labels of the data, we can evaluate the method in the same way as is done in classification (supervised). A data point is considered to be well classified if the cluster to which it was assigned by the network corresponds to its actual label. Consequently, confusion matrices are used to show the results. The sensitivity (true positive rate) and the specificity (false positive rate) of every single cellular type can be derived from the confusion matrices. 

The results are shown for three different cases. The first case, referred to as “case-1”, consists of using the network without the proposed techniques for assuring a better reconstruction, which means that we rely only on the reconstruction loss in order to assure a better local preservation, as proposed in [32,33]. The second case, referred to as “case-2”, consists of using only the pooling indices, as shown in the network depicted in Figure 3. The final case, referred to as “case-3”, consists of using the pooling indices and the copy-and-concatenation techniques at the same time, as illustrated in the network shown in Figure 4.

### 3.2. Results for Case-1

**Figure 6:**
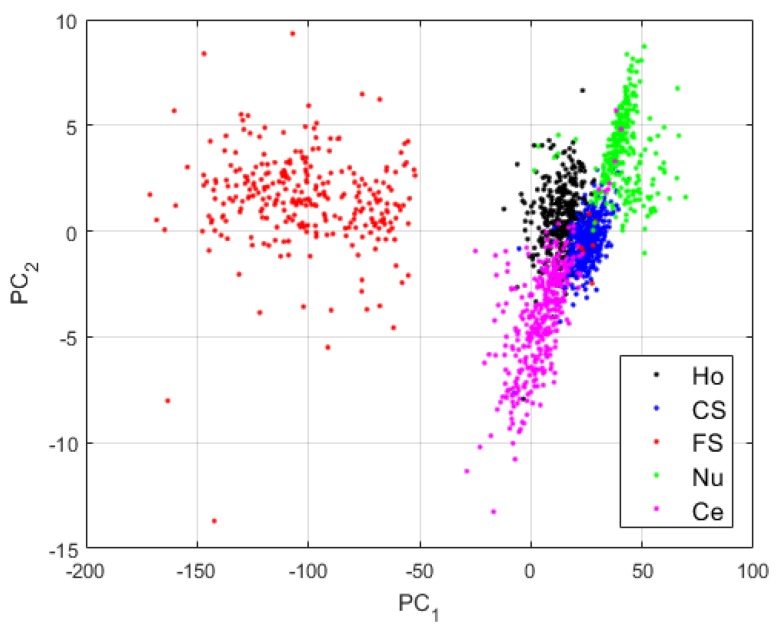
Visualization of the features learned by the DCAE in case-1. “Ho”, “CS”, “FS”, “Nu”, and “Ce” represent the homogeneous, the coarse speckled, the fine speckled, the nucleolar and the centromere cells, respectively. The percentages of the variance explained are, respectively, 99.23% and 0.42% for the first and second principal components.

For the first case, we use the network as defined in Table 1, without applying the techniques proposed here for assuring a good local preservation of the pixels, thus, a better reconstruction. After the pre-training (γ=0), we use the pre-trained DCAE in order to perform the global learning (γ>0). Note that the features learned by the DCAE, as is the case for the clusters’ centroids, are 512-dimensional vectors, as shown in Table 1. For visualizing these high-dimensional vectors, we have applied principal component analysis (PCA) [48]. For all the features’ visualization shown here, PC_1_ and PC_2_ are, respectively, the first and second axis of the PCA-space. The projections of the clusters learned by the case-1 DCAE are shown in Figure 6. 

The first observation from the projections shown in Figure 6 is that the network has clearly learned to distinguish the different cellular types. Two main clusters are visible in Figure 6: the cluster formed by the fine speckled cells and that formed by the remaining cells. The fine speckled cells, as we can see in Figure 5, are remarkably distinguishable from the others. The fine speckled cluster itself contains two subgroups, which represent the two different levels of fluorescence illumination (intensity levels). If we can expect from these features that the majority of the fine speckled will be well classified (assigned to the cluster that corresponds to their true labels), the situation is more complicated for the other clusters that share many similarities. 

**Figure 7:**
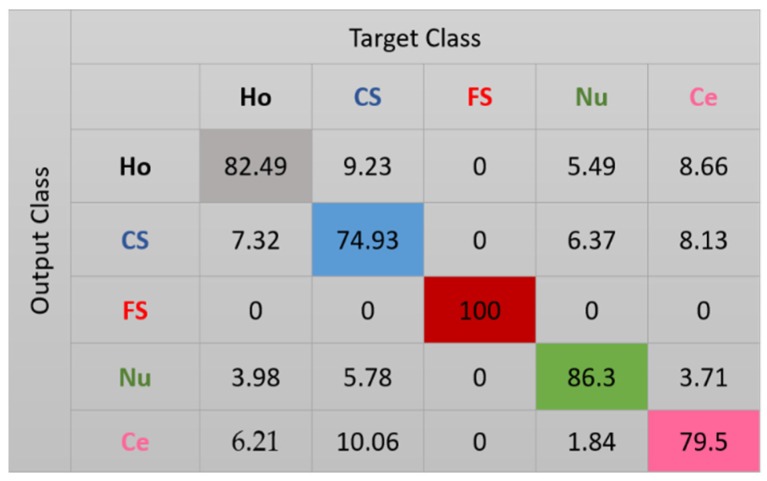
Confusion matrix for case-1. “Ho”, “CS”, “FS”, “Nu”, and “Ce” represent homogeneous, coarse speckled, fine speckled, nucleolar, and centromere cells, respectively.

The results for the case-1 network are shown in the confusion matrix depicted in Figure 7. As expected, all the fine speckled cells were assigned to the right cluster, that is, the one that corresponds to their true label. The remaining cells all suffer from a lack of clear distinguishability between them, which significantly decreases the accuracy of the cluster’s assignment. Note that, as for the projections shown in Figure 6, every cluster is represented by its corresponding color in Figure 7: black for homogeneous, blue for coarse speckled, red for fine speckled, green for nucleolar, and magenta for centromere. Major confusions in Figure 7 concern the coarse speckled and the centromere cells, whose assignment accuracies are 74.93% and 79.5%, respectively. The total accuracy of the case-1 DCAE is about 84.64%. 

### 3.3. Results for Case-2

**Figure 8:**
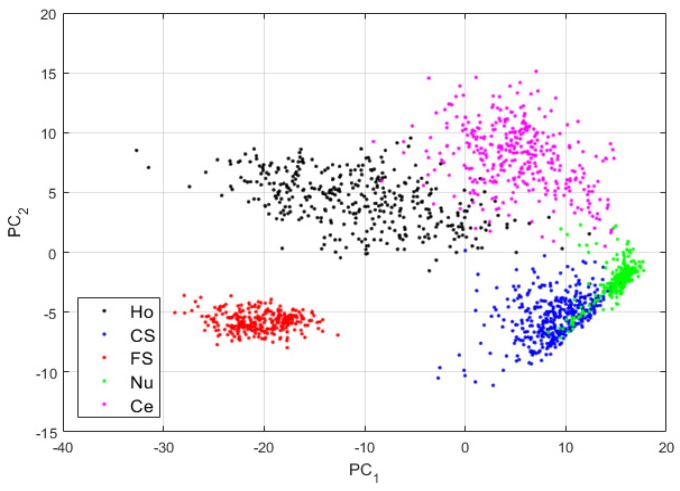
Visualization of the features learned by the DCAE in case-2. “Ho”, “CS”, “FS”, “Nu”, and “Ce” represent the homogeneous, coarse speckled, fine speckled, nucleolar and centromere cells, respectively. The percentages of the variance explained are, respectively, 83.06% and 16.38% for the first and second principal components.

For case-2, we use the network as depicted in Figure 3. The features are shown in Figure 8. 

**Figure 9:**
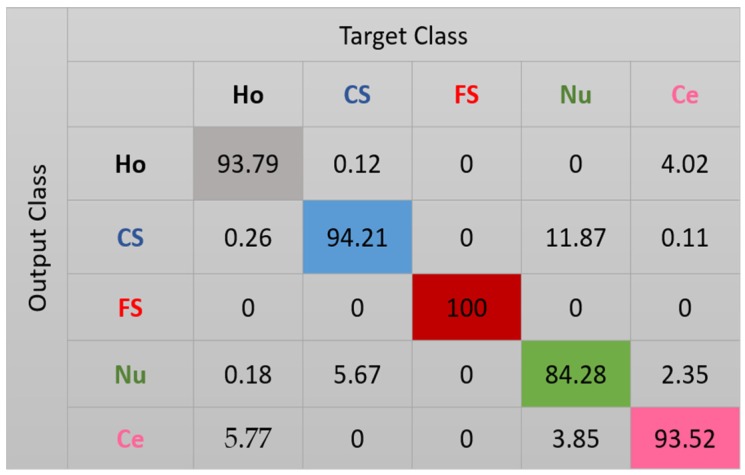
Confusion matrix for case-2. “Ho”, “CS”, “FS”, “Nu”, and “Ce” represent homogeneous, coarse speckled, fine speckled, nucleolar and centromere cells, respectively.

By comparing the projections shown in Figure 6 and those shown in Figure 8, we can note how the four other clusters have become distinguishable. The fine speckled cells are still clustered to their own sub-space, as for the projections in case-1. This shows that when the reconstruction process from the DCAE tends to preserve the local structure of the original images in the best way possible, the features learned by the DCAE are also better. The results for the cluster’s assignment are shown in the confusion matrix in Figure 9.

In Figure 9, we see how most of the confusions between the cells have completely disappeared. The remaining wrong assignments concern mostly the nucleolar cells. As we can see in the projections shown in Figure 8, the nucleolar cells (shown in green) tend to share the same clustering subspaces with the coarse speckled and the centromere cells. This is explained by the similarities shared by these three cellular types in terms of the shape and intensity, as we can note in Figure 5. Specifically, 11.87% of the nucleolar cells are clustered as coarse speckled. In Figure 8, the features from the nucleolar and coarse speckled (green and blue) are largely mixed. Moreover, 5.67% of the coarse speckled are misclassified as nucleolar. Apart from that, the clustering accuracy increases from 84.64% (for case-1) to 93.16%, which clearly indicates that the more we can encourage the network to preserve the local structure of the original images, the more the features learned by the network will contain the distinctive characteristics of the cellular images.

### 3.4. Results for Case-3

The third case consists of using the network as designed in Figure 4, where the pooling indices storage technique is mixed with the copy-and-concatenation process. The copy-and-concatenation operations allow the network’s decoder to retrieve the entire spatial information lost during the down-sampling operated in the encoder. When mixed with the pooling indices storage, not only are the local structures (close neighborhood) of the original pixels well preserved, but the global spatial information, concerning the whole image, is also retrieved during the reconstruction. 

**Figure 10:**
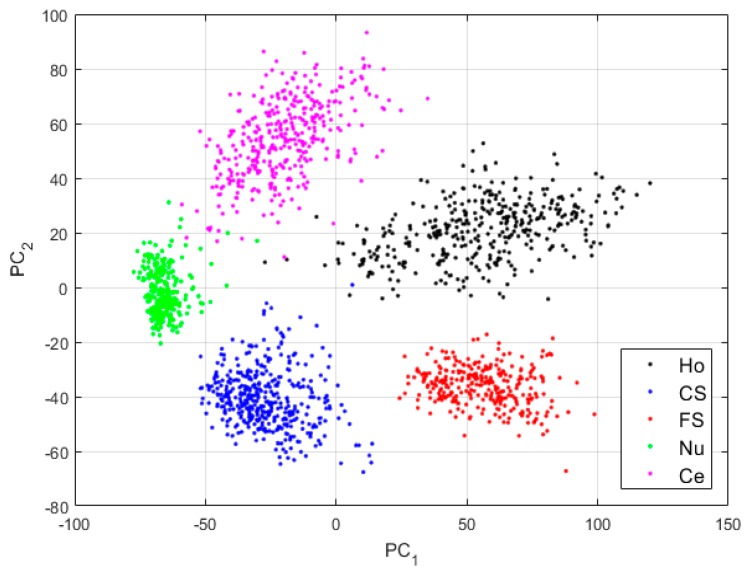
Visualization of the features learned by the DCAE in case-3. “Ho”, “CS”, “FS”, “Nu”, and “Ce” represent homogeneous, coarse speckled, fine speckled, nucleolar, and centromere cells, respectively. The percentages of the variance explained are, respectively, 56.86% and 33.61% for the first and second principal components.

**Figure 11:**
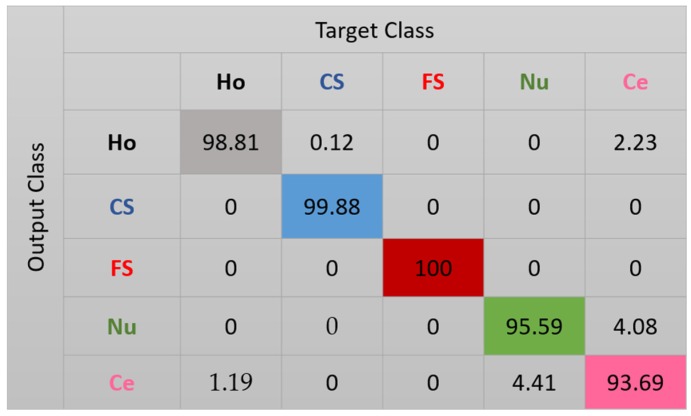
Confusion matrix for case 3. “Ho”, “CS”, “FS”, “Nu”, and “Ce” represent homogeneous, coarse speckled, fine speckled, nucleolar, and centromere cells, respectively.

The features for this case are shown in Figure 10. We can note how, compared with the projections in Figure 8, the clusters continue to be more precise. Some of the nucleolar cells continue to be mixed with the centromere cells but, globally, the confusions between the cells are significantly diminished. The cluster’s assignment results are shown in detail in the confusion matrix depicted in Figure 11. As we can observe, most of the misclassifications have disappeared. The centromere cells are still mixed with some homogeneous (2.23%) and nucleolar (4.08%) cells. The most outstanding improvement comes from the nucleolar cells. The totality of the confusion between the nucleolar and the coarse speckled cells has vanished, with only some misclassification remaining between the nucleolar and centromere cells. The total accuracy of the clustering assignment is 97.59% for the results shown in Figure 11.

**Figure 12:**
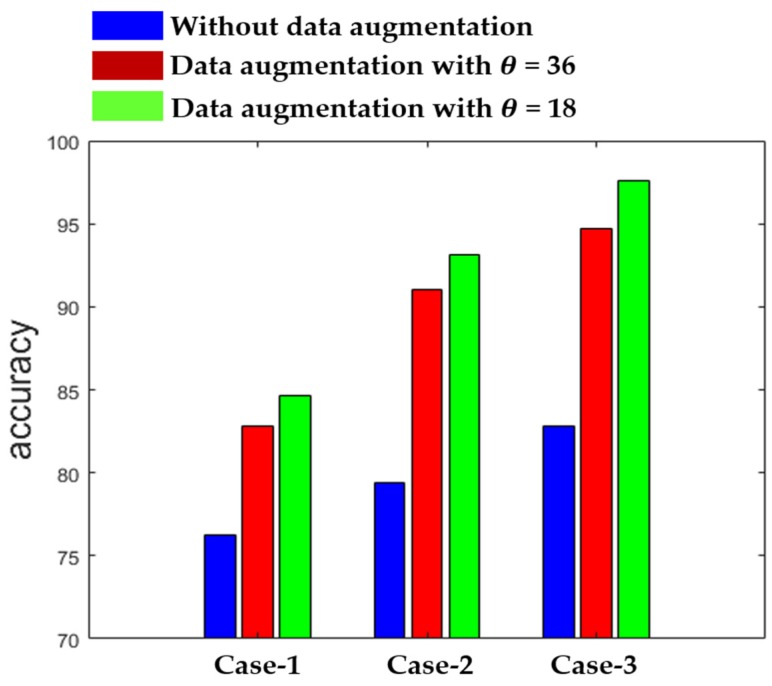
Clustering accuracy of the three networks.

Figure 12 shows the summary of the results obtained by the three different networks in terms of the accuracy. The results in Figure 12 also demonstrate the effectiveness of using data augmentation when the training dataset is relatively small. As we can observe, the results remain poor with the three networks when we use the original data without any augmentation during the training. On the other hand, we can see how the accuracy is improved when data augmentation is applied. We show the results for θ = 36, which means that the rotation is undertaken with a step of 36°, increasing the number of training images by a factor of 10, and for θ = 18, which increases the training images by a factor of 20. Each of the three networks provides the best results with θ = 18, as shown in Figure 12. 

**Figure 13:**
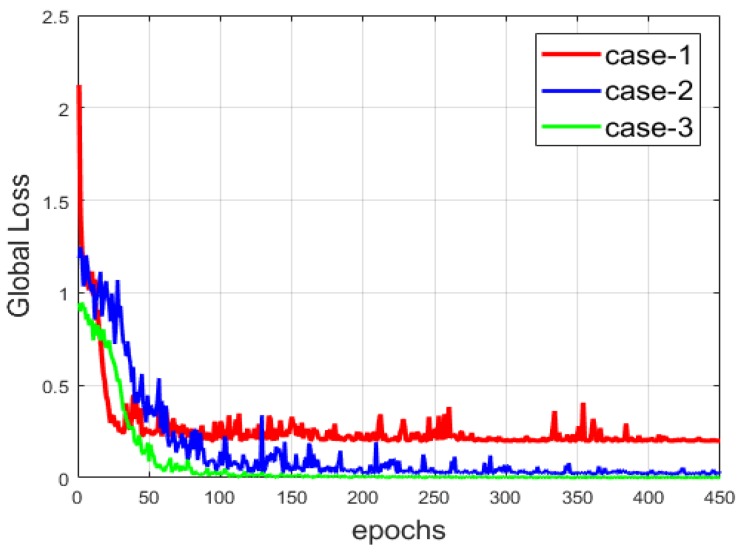
The global loss of the three networks.

Another comparison between the three networks is provided in Figure 13, where we show the evolution of the global loss, which encapsulates the reconstruction and the clustering losses. Among the three cases, the network from case-3 provides the most minimal loss, since the copy-and-concatenation process alleviates the reconstruction process by allowing the network to preserve, at the best way possible, the local structure of the data. We can observe that the loss is smoother than that of the two other cases. The local structure preservation permits a faster reconstruction compared to the others. Note that the loss is also consequently diminished in case-2. With the results shown in both Figure 12 and Figure 13, we can note the improvement that occurs when the reconstruction is made in the best way possible (case-2 and case-3).

Another finding concerns the parameter γ that controls the balance between the reconstruction and the clustering losses. As mentioned before, the best results were obtained with the value of 0.1. We found that the accuracy decreases every time the coefficient γ increases, which means that when the clustering loss tends to overshadow the reconstruction loss, the features lose their distinctive characteristics, thereby encouraging misclassification during the assignment. In addition to the results demonstrated above, this fact also corroborates the assumption that the preservation of the local structure of the images helps to produce better features.

**Figure 14:**
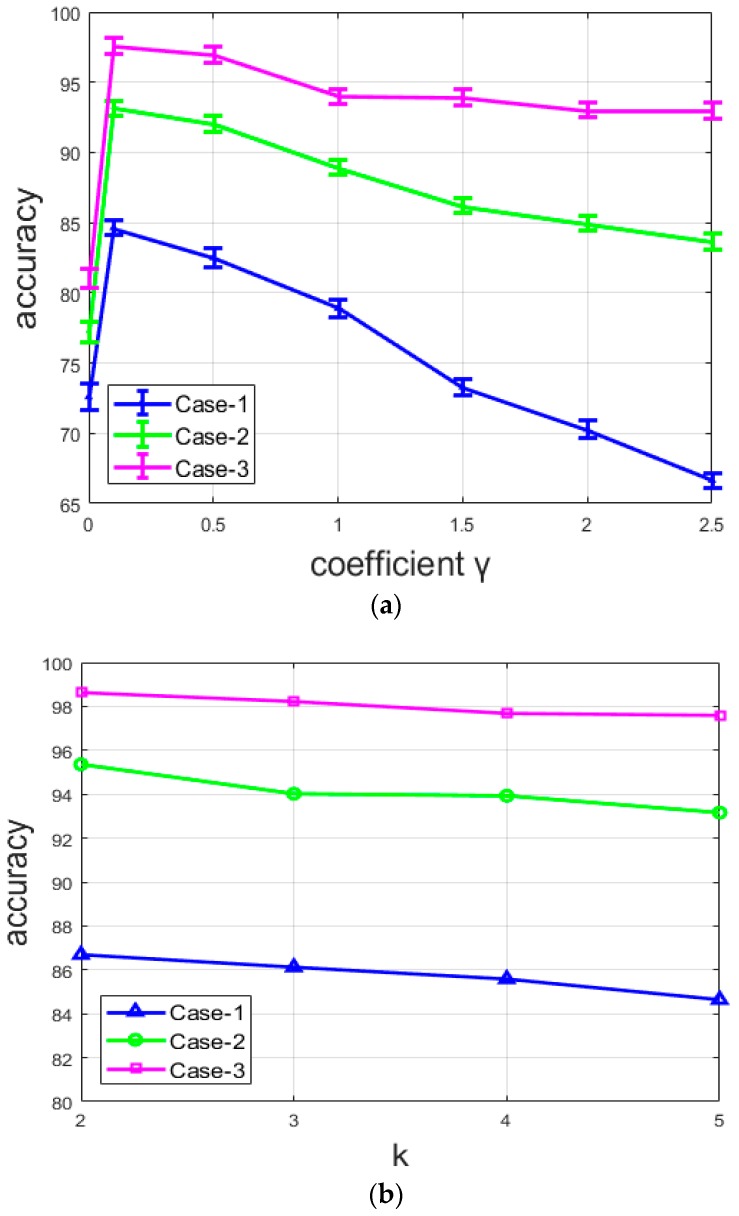
Accuracy of the three networks: (a) with different values of coefficient γ, and (**b**) with different values of *k* (number of clusters).

Figure 14a shows the variations of the clustering accuracy with different values of the coefficient γ for the three different networks. Note that, here, the situation where γ=0, as explained above, corresponds to the pre-training of the DCAE, where only the reconstruction loss is used in order to generate the initial features that will be used to compute the initial centroids. We can note that, for all the networks, the accuracy for the pre-training (γ=0) is very low. With γ>0, all the networks provide their best results with the value of 0.1 and their accuracy starts to decrease with every γ>0.1. The most important point is that the accuracy decreases differently for the three networks. It decreases rapidly with the network from case-1, slightly more slowly with that from case-2, and seems to be stable with the network from case-3. For case-1, there is an important decline of the accuracy when the coefficient γ>0.1. This network does not contain any element in its design that can lessen the loss of the local structure, except, of course, the reconstruction loss. It clearly appears that the clusters lose their efficiency when the clustering loss completely overshadows the reconstruction loss. On the other hand, the case-2 and case-3 networks are purposely designed to minimize the loss of the spatial details and preserve the local structure of the original images. Both suffer a decrease of the accuracy with γ>0.1, but the reduction is minimized.

From Figure 14a, we can also remark that the accuracy of the network is not highly dependent on the value of the coefficient γ for the case-3 network. Even though there is an evident decrease, the variation is not particularly noticeable compared to the two other cases. This is because the two combined techniques used for case-3 allow the network to continue to assure a better reconstruction even when the clustering loss tends to overshadow the reconstruction loss. With these results, we can affirm that the quality of the reconstruction process affects the quality of the produced features, and thus, the final clustering’s accuracy. In Figure 14b, we show the variation of the accuracy by changing the number of clusters. Note that the variation is small because the value of *k* is also small. Therefore, as we can clearly observe in Figure 14b, there is no noticeable change when the number of clusters changes.

In Table 2, we show the results of the different methods in the literature. We separate the methods into three groups: the supervised learning handcrafted features, the supervised deep learning methods, and the proposed unsupervised deep learning method. Supervised learning methods based on handcrafted features achieved accuracies of, respectively, 80.90%, 82.50%, and 85.71%. The method in [22], which was one of the first to apply deep learning for HEp-2 cell image classification, attained an accuracy of 86.20%. Note that the datasets available at that period were not diversified enough to perform well with deep learning. The deep learning-based method in [29] had a quasi-identical structure to the one used in [22]. Unlike in [22], they applied many different techniques for data augmentation, allowing them to achieve an accuracy of 88.37%. Table 2, in the last three lines of the “supervised deep learning” partition, shows the performance of the actual state-of-the-art methods. Of course, as discussed before, each of these utilized the supervised learning approach.

Table 2 shows that the case-1 network performs at the same level as the hand-crafted features. When we apply the proposed approach (case-2 and case-3), the proposed method performs at the same level as (case-2), or even slightly better than (case-3), the state-of-the-art supervised deep learning methods. 

The ICPR 2016 dataset [37] was also used in order to test the proposed method. This dataset has a far bigger number of data points (13,596) compared to the first dataset and seems to be much easier to handle using deep learning-based methods. Cross-validation was performed using the protocol defined in [22]. A portion of 80% of the data was used for training and validation (using a 64%/16% split) and the remaining 20% was utilized for testing. We applied data augmentation in the same way as described previously. 

Compared to the first dataset, the handcrafted features perform poorly. The reason is that the large number of images from this dataset provides a strong learning capability for the deep learning methods while it brings more complexity to the handcrafted methods. For this dataset, we show the results just as they were reported by the authors in their works. We experimented only with the handcrafted features’ works because all were proposed at a time when this dataset was not available. The results are shown in Table 3.

Note that, as we can observe from Table 3, all of the state-of-the-art supervised deep learning methods perform similarly on this dataset. Our method (case-2 and case-3) also performs at the same level as the supervised learning methods in terms of accuracy, but with the advantage of being entirely unsupervised. In addition, the F1-scores for the three cases are 90.57, 95.09, and 98.74 for case-1, case-2, and case-3, respectively, which is at the same level of performance as the supervised learning methods.

## 4. Conclusions

HEp-2 cell classification is one of the most important steps for the automated diagnosis of autoimmune diseases. The majority of the published works in this topic and, particularly, the state-of-the-art methods, prefer to address this problem with the use of the supervised learning technique. As the first contribution of the present study, we proposed a classification method based on a strictly unsupervised learning paradigm; that is, both feature extraction and discrimination are performed using an unsupervised learning approach. 

We embedded a clustering layer in the DCAE in such a way that the network will perform feature extraction and learn how to cluster the produced features at the same time. As the second and principal contribution, we designed different networks that are capable of preserving in the best way possible the local structure of the original images in order to assure a fair reconstruction. We demonstrated that the quality of the reconstruction process directly affects the quality of the produced features. The discrimination potentiality of the features produced by our networks was tested on two publicly available datasets and the results show that the proposed method performs as well as the existing state-of-the-art supervised deep learning methods. 

In terms of accuracy, the proposed method attains the same level as the supervised learning methods. In addition, our approach has the advantage of not requiring the use of a single labelled data point during the training of our networks. In the near future, when huge amounts of data will be used, the proposed approach will have the advantage of avoiding the burden of manually labelling images. 

## Figures and Tables

**Table 1:** Architecture of the deep convolutional autoencoder (DCAE).

Layer	Filter Size	#Feature Maps	Stride	Padding	Output
Input	-	1	-	-	112 × 112
Conv 1	3 × 3	32	1	1	112 × 112
Pool 1	2 × 2	32	2	0	56 × 56
Conv 2	3 × 3	64	1	1	56 × 56
Pool 2	2 × 2	64	2	0	28 × 28
Conv 3	3 × 3	128	1	1	28 × 28
Pool 3	2 × 2	128	2	0	14 × 14
Conv 4	3 × 3	256	1	1	14 × 14
Pool 4	2 × 2	256	2	0	7 × 7
Conv 5	7 × 7	**512**	1	1	1 × 1
Conv 6	7 × 7	256	1	0	7 × 7
Unpool 4	2 × 2	256	2	0	14 × 14
Conv 7	3 × 3	128	1	1	14 × 14
Unpool 3	2 × 2	128	2	0	28 × 28
Conv 8	3 × 3	64	1	1	28 × 28
Unpool 2	2 × 2	64	2	0	56 × 56
Conv 9	3 × 3	32	1	1	56 × 56
Unpool 1	2 × 2	32	2	0	112 × 112
Conv 10	3 × 3	1	1	1	112 × 112

**Table 2:** Comparative study for the SNPHEp-2 dataset.

Method	Description	Accuracy
Supervised learning Hand-crafted features	Texture features + SVM [49]	80.90%
	DCT features + SIFT + SVM [5]	82.50%
	LBP + SVM [6]	85.71%
Supervised Deep Learning	Simple CNN [22]	86.20%
	Simple CNN [29]	88.37%
	CNN with Deep Residual Inception Module [23]	95.61%
	CNN using Cross-modal transfer learning [27]	95.99%
	CNN with a Deep-Cross Residual Module [28]	96.26%
Unsupervised Deep Learning	DCAE with an embedded clustering layer (case-1)	84.64%
	DCAE with an embedded clustering layer (case-2)	93.16%
	DCAE with an embedded clustering layer (case-3)	97.56%

**Table 3:** Comparative study for the ICPR 2016 dataset.

Method	Description	Accuracy
Supervised Learning Hand-crafted features	Texture features + SVM [49]	71.63%
	DCT features + SIFT + SVM [5]	74.91%
	LBP + SVM [6]	79.44%
Supervised Deep Learning	Simple CNN with 5 layers [22]	97.24%
	VGG-like network [29]	98.26%
	CNN with a Deep Residual Inception Module [23]	98.37%
	CNN with Cross-modal transfer learning [27]	98.42%
	CNN with the use of a Deep-Cross Residual Module [28]	98.82%
Unsupervised Deep Learning	DCAE with an embedded clustering layer (case 1)	89.13%
	DCAE with an embedded clustering layer (case 2)	94.48%
	DCAE with an embedded clustering layer (case 3)	98.51%
